# The Effect of Short-Term Consumption of Lactic Acid Bacteria on the Gut Microbiota in Obese People

**DOI:** 10.3390/nu14163384

**Published:** 2022-08-18

**Authors:** Inna Burakova, Yuliya Smirnova, Mariya Gryaznova, Mikhail Syromyatnikov, Pavel Chizhkov, Evgeny Popov, Vasily Popov

**Affiliations:** 1 Laboratory of Metagenomics and Food Biotechnology, Voronezh State University of Engineering Technologies, 394036 Voronezh, Russia; 2 Department of Genetics, Cytology and Bioengineering, Voronezh State University, 394018 Voronezh, Russia

**Keywords:** microbiome, obesity, lactic acid bacteria, *16S rRNA*, sequencing

## Abstract

Obesity is a problem of modern health care that causes the occurrence of many concomitant diseases: arterial hypertension, diabetes mellitus, non-alcoholic fatty liver disease, and cardiovascular diseases. New strategies for the treatment and prevention of obesity are being developed that are based on using probiotics for modulation of the gut microbiota. Our study aimed to evaluate the bacterial composition of the gut of obese patients before and after two weeks of lactic acid bacteria (*Lactobacillus acidophilus*, *Lactiplantibacillus plantarum*, *Limosilactobacillus fermentum*, and *Lactobacillus delbrueckii*) intake. The results obtained showed an increase in the number of members of the phylum Actinobacteriota in the group taking nutritional supplements, while the number of phylum Bacteroidota decreased in comparison with the control group. There has also been an increase in potentially beneficial groups: *Bifidobacterium*, *Blautia*, *Eubacterium*, *Anaerostipes*, *Lactococcus*, *Lachnospiraceae ND3007*, *Streptococcus*, *Escherichia-Shigella*, and *Lachnoclostridium*. Along with this, a decrease in the genera was demonstrated: *Faecalibacterium*, *Pseudobutyrivibrio*, *Subdoligranulum*, *Faecalibacterium*, *Clostridium sensu stricto 1* and *2*, *Catenibacterium*, *Megasphaera*, *Phascolarctobacterium*, and the *Oscillospiraceae NK4A214* group, which contribute to the development of various metabolic disorders. Modulation of the gut microbiota by lactic acid bacteria may be one of the ways to treat obesity.

## 1. Introduction

The gut microbiome strongly contributes to the overall health of the host including the risk of developing obesity. Although our knowledge about the composition of the human microbiome is evolving quite rapidly, there are many questions about the metabolic mechanisms and functionality of the human microbiome. It is known that probiotics have a positive effect in the context of various diseases, particularly those associated with gastroenterology [1]. A deeper understanding of the effect of probiotics on the composition of the microbiome should help in the treatment and prevention of various diseases.

The prevalence of obesity worldwide continues to rise [2]. Even though obesity is a metabolically complex and multifactorial disease, aberrations in the composition of the gut microbiome make a great contribution to the formation of this pathology [3,4]. There are many studies describing dysbiosis in people with obesity, characterized by less diversity [5,6,7]. Dysbiosis likely contributes to the development of obesity and metabolic complications through various mechanisms including changes in the regulation of energy, gut hormones, pro-inflammatory mechanisms, and others [8].

Since weight loss drugs often have specific toxic side effects, it is necessary to look for more moderate and effective substitutes. Some research suggests that the gut microbiota is directly linked to obesity and taking probiotics may have a positive impact on weight loss [9,10].

Lactic acid bacteria are often used as probiotics. Many species of lactic acid bacteria can boost nutrient absorption, improve the functioning of the gastrointestinal tract, lower serum cholesterol levels, prevent obesity, inhibit the growth of putrefactive bacteria in the intestine, and improve immune function [11,12,13,14].

*Lactobacillus acidophilus* is one of the main commercially important species of lactic acid bacteria used as a probiotic [15]. *L. acidophilus* promotes nutrient absorption, has a beneficial effect on digestive functions, and prevents diarrhea [16].

There is growing evidence that *L. plantarum* may benefit human health [17]. For example, there is evidence that *L. plantarum* can lower cholesterol levels in adults with hypercholesterolemia, potentially reducing the risk of coronary heart disease [18]. Additionally, *L. plantarum* often exhibits the broadest ability to inhibit pathogen growth among the *Lactobacilli* species [19,20].

*L. fermentum* is one of the most promising probiotics. It is often used as a typical reference species in comparative investigations with different probiotics due to its health benefits [21]. There are studies that describe the potential beneficial role of *L. fermentum* in various diseases such as intestinal inflammation [22], respiratory infections [23], and hepatic injury [24].

Despite the reported beneficial properties of these lactic acid bacteria species, supporting studies are needed to further understand the role of these probiotics in modulating gut microbial populations.

It is possible that restoring the gut microbiome to a healthy state could improve the conditions associated with obesity. However, the role of probiotics in the prevention and treatment of obesity has not yet been fully explored.

The purpose of this work was to study the effect of probiotic intake on the composition of the gut microbiome in obese people.

## 2. Materials and Methods

### 2.1. Experiment Design

At the beginning of the study, each patient completed a questionnaire that included questions about age, nutrition, physical activity, a history of infectious and non-infectious pathologies, taking medications including hormonal and antibacterial drugs (for the last 6 months), and there were clarifications on anthropometric indicators (height; weight for calculating body mass index (BMI)) and waist circumference (OT). Based on the results of the survey, eight patients were selected, who were subsequently included in the group with diagnosed obesity.

At the beginning of the study, each of the eight patients had a fecal sampling, approximately 20 ± 5 g. Then, each patient consumed 55 g of the supplement, which consisted of lactic acid bacteria, with food for two weeks. During this period, patients did not change their usual diet in any way. At the end of the 2-week supplementation, approximately 20 ± 5 g of feces were again collected from each patient for microbiome profiling.

The study was conducted according to the guidelines of the Declaration of Helsinki and approved by the Institutional Ethics Board of Voronezh State University (Protocol N42-03, 7 March 2022). Before the beginning of the study, all patients gave their written consent to the use of anonymized personal data for scientific purposes.

### 2.2. Dietary Supplement of Lactic Acid Bacteria

Probiotic bacteria were obtained from LLC “White Lily” (Voronezh, Russia). After purification, the bacterial cultures received the following numbers: *Lactobacillus delbrueckii subsp. bulgaricus VSUET15*; *Lactiplantibacillus plantarum VSUET13*; *Lactobacillus acidophilus VSUET12*; and *Limosilactobacillus fermentum VSUET14* for further placement in the Microbiological Museum of the Voronezh State University of Engineering Technologies.

For subsequent work, the bacteria were added to 100 mL of sterilized skimmed milk. To improve the fermentation process in the mixture of bacteria, *Streptococcus thermophilus* was added. The activation of microbial cells of the concentrate occurred due to mixing and further 4 h exposure at a constant temperature of 37 ± 1 °C, cyclically stirring (shaking) for a uniform distribution of bacterial cells in the mixture. Then, the produced concentrate was immediately added to pasteurized (92 ± 2 °C, exposure 2–8 min) and chilled (37–42 °C) skimmed milk with constant stirring. The density of microorganisms in the finished product was adjusted to 10^8^ CFU/mL. Subsequently, the cultures were kept at a temperature of −u °C for no more than three days. The viable cells of lactic acid bacteria were accounted on MRS agar.

### 2.3. 16S rRNA Gene Sequencing

A commercial Quick-DNA Fungal/Bacterial Microprep Kit (Zymo Research; Irvine, CA, USA) was used to obtain a DNA product from a biomaterial.

Analysis of the bacterial diversity of the intestinal microbiome by sequencing based on the Ion Torrent PGM platform was carried out for the V3 hypervariable region of the *16S rRNA* gene.

For further PCR analysis, two universal primers 337F and 518R (the sequence of primers is presented in Table 1) and a commercial set of the 5X Screen Mix-HS Master Mix Kit (Eurogen, Moscow, Russia) were used.

The PCR protocol is presented in Table 2.

We used AMPureXP magnetic beads to purify PCR-derived amplicons (Beckman Coulter, Brea, CA, USA) for further preparation of the sequencing libraries. This is a necessary step for preparing high-quality sequencing libraries.

For the preparation of sequencing libraries, a commercial NEBNext Fast DNA Kit (New England Biolabs, Ipswich, MA, USA) was used in the work according to the method provided by the manufacturer. In further work, the resulting library was barcoded with a commercial NEXTflex Kit (Ion Torrent; 64 adapters; PerkinElmer, Inc., Waltham, MA, USA). Then, another cleaning of the finished libraries was carried out with AMPureXP magnetic particles (Beckman Coulter, Brea, CA, USA). The Ion Torrent PGM platform was used for sequencing. Libraries were loaded onto an Ion 318™ Chip v2 BC using the standard protocols of the Ion PGM Hi-Q View OT2 Kit for further enrichment using emulsion PCR and the Ion PGM Hi-Q View Sequencing Kit for post-processing and loading libraries onto the chip as well as for running the instrument itself (ThermoFisher Scientific, Madison, WI, USA).

### 2.4. Statistical Analysis

The obtained analysis results were processed using the R programming language in the R Studio environment (V SEARCH v software.2.8.2; version 1.1.414 ©2009-2018 RStudio Inc., RStudio PBC, Boston, MA, USA).

At the initial stage, BAM files were generated for each sample, which were later modified into the FastQ format based on the fileexporterwin plugin.

To exclude low-quality reads from the subsequent analysis, we used the maximum expected error threshold, all reads for which values of less than 1.0 were eliminated (DADA2 package). In addition, the reads were truncated to an optimized total size, followed by demultiplexing. After all the manipulations, the reads were dereplicated and, as a result, all identical reads were combined into unique sequences. Then, based on the UNOISE2 algorithm, we formed operational taxonomic units (OTUs).

Generic identification was carried out using version 132 of the SILVA database (https://www.arb-silva.de/, accessed on 14 March 2022). The taxonomy was determined relative to the peak limit of identity with variant amplicon sequences corresponding to 100%. All raw sequences obtained from the analyses for each sample are available in the BioProject repository (Bio Project:PRJNA862929).

Statistical analysis of the sequencing data was carried out on the basis of GraphPad Prism 9 software (GraphPad, San Diego, CA, USA). The statistical significance of differences in the microbiome profiles of patients was achieved using the Mann–Whitney U test. The average abundance of individual taxa was expressed as the mean ± standard error of the mean (SEM). The intra-sample alpha diversity score was expressed using Shannon’s test.

## 3. Results

In total, 128,390 unique reads were obtained during sequencing, corresponding to 103 bacterial genera (Table S1).

**Figure 1:**
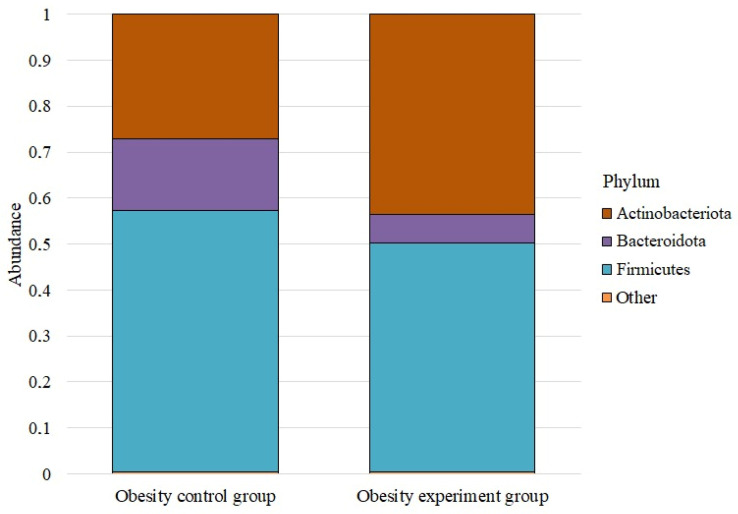
The average abundance of phyla in the studied groups.

We analyzed the microbiome composition at the phylum level for the study groups (Figure 1).

The phylum Firmicutes dominated in both cases. Before taking lactic acid bacteria, its abundance was 0.569 ± 0.072; after taking it, it decreased to 0.498 ± 0.048. The phylum Actinobacteriota was the next most numerous both before and after taking the lactic acid bacteria supplement. In the control group, its abundance was 0.272 ± 0.025, while in the experimental group, it was 0.436 ± 0.036. The abundance of the phylum Bacteroidota before taking the supplement was 0.155 ± 0.078; after, it was—0.061 ± 0.020. Proteobacteria were next, with an abundance of 0.003 ± 0.001 and 0.005 ± 0.001, respectively. The number of Verrucomicrobiota before taking lactic acid bacteria was 0.0008 ± 0.001; after, it was — 0.0002 ± 0.001. The Desulfobacterota phylum was the least numerous; in both groups, its abundance was less than 0.005.

**Figure 2:**
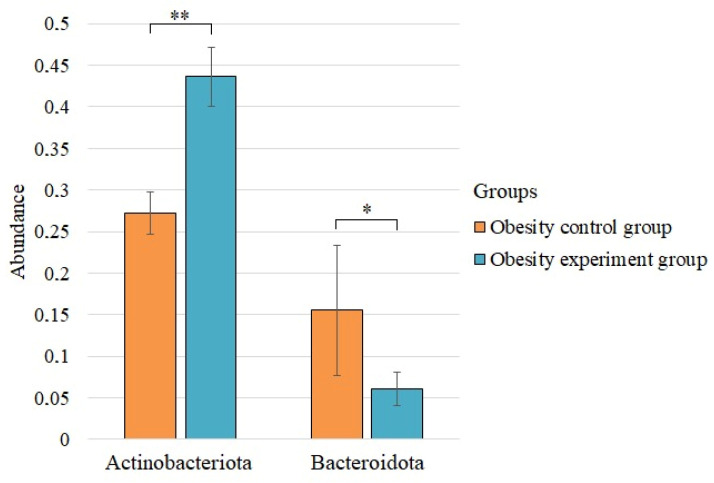
The statistically significant differences in the phylum composition of the control group of obesity and the group of patients with obesity after taking lactic acid bacteria. * *p* < 0.05; ** *p* < 0.01.

We found statistically significant differences before and after the intake of lactic acid bacteria for the two phyla (Figure 2).

The abundance of the phylum Actinobacteriota increased significantly after supplementation with lactic acid bacteria, while the abundance of Bacteroidota decreased.

**Figure 3:**
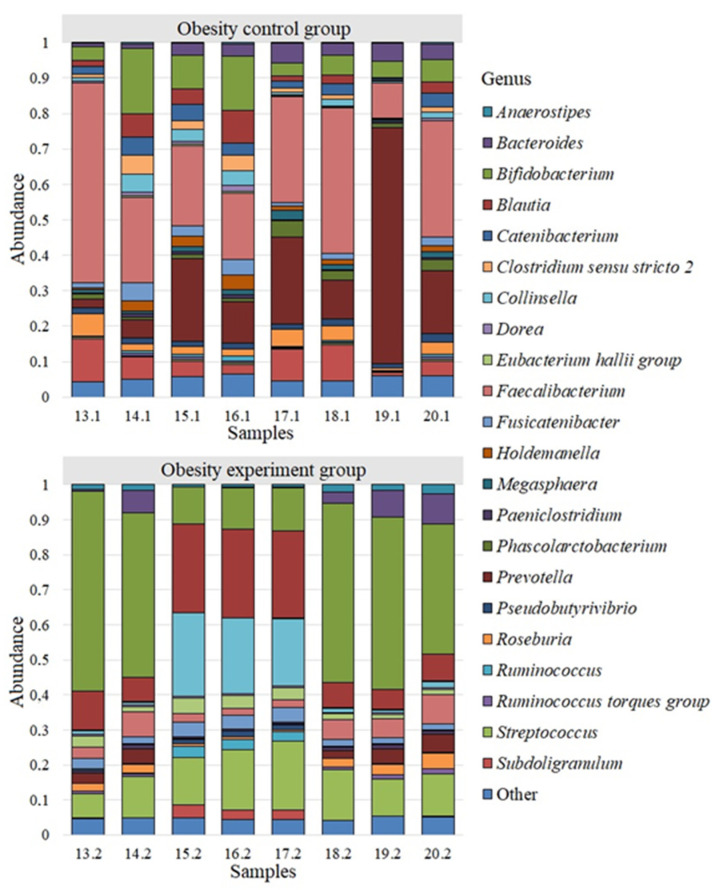
The microbiome of obesity patients before and after the intake of lactic acid bacteria.

Bacterial genera whose abundance was less than 0.005 were grouped into “Other Genus”. We analyzed the differences among all identified genera of bacteria for each study group. Twenty-two of the most common bacterial genera were identified for the group of obese patients participating in a study evaluating the effects of a nutritional supplement containing lactic acid bacteria on the gut microbiome composition (Figure 3).

We detected a different microbiome landscape in the group of patients with obesity. *Faecalibacterium* with a population of 0.294 ± 0.05 was also the most common genus. The next largest genera were *Prevotella*—0.203 ± 0.072; *Bifidobacterium*—0.084 ± 0.019; *Subdoligranulum*—0.062 ± 0.014; *Blautia*—0.037 ± 0.011; *Bacteroides*—0.035 ± 0.006; *Roseburia*—0.032 ± 0.006; *Catenibacterium*—0.031 ± 0.005; *Fusicatenibacter*—0.024 ± 0.006; *Collinsella*—0.022 ± 0.006; *Clostridium sensu stricto 2*—0.022 ± 0.006; *Phascolarctobacterium*—0.020 ± 0.005; *Holdemanella*—0.019 ± 0.005; *Pseudobutyrivibrio*—0.017 ± 0.001; *Megasphaera*—0.013 ± 0.002. The average abundance of other genus in the group of patients with obesity was less than 0.001.

We observed a microbiome landscape dominated by the genus *Bifidobacterium*—0.346 ± 0.070 after a two-week intake of lactic acid bacteria in obese patients. The next largest genera were *Blautia*—0.142 ± 0.033; *Streptococcus*—0.132 ± 0.014; *Collinsella*—0.088 ± 0.038; *Faecalibacterium*—0.046 ± 0.009; *Bacteroides*—0.034 ± 0.013; *Fusicatenibacter*—0.028 ± 0.004; *Eubacterium hallii group*—0.026 ± 0.004; *Prevotella*—0.024 ± 0.007; *Roseburia*—0.021 ± 0.005; *Anaerostipes*—0.014 ± 0.003; *Subdoligranulum*—0.012 ± 0.006; *Ruminococcus*—0.011 ± 0.005. The number of other bacterial genera was less than 0.010.

**Figure 4:**
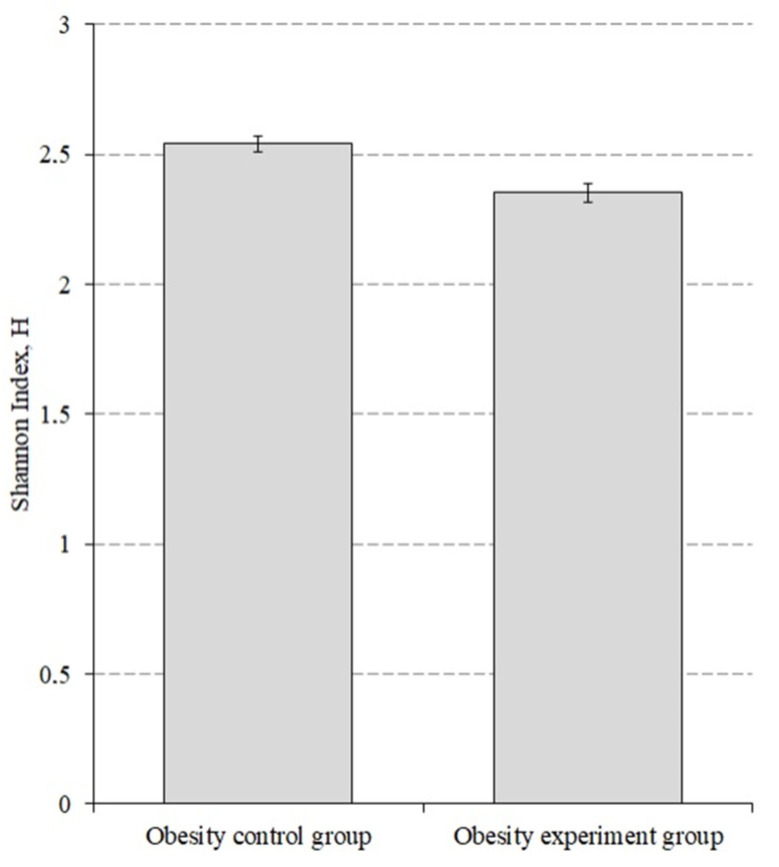
The alpha diversity index for the obesity patients before and after the intake of lactic acid bacteria. *p* < 0.001.

We also evaluated the alpha diversity before and after lactic acid bacteriaintake by obesity group of patients (Figure 4).

The alpha diversity index before taking the lactic acid bacteria was 2.540 ± 0.031, while after taking it decreased to 2.351 ± 0.035 (*p* < 0.001).

**Figure 5:**
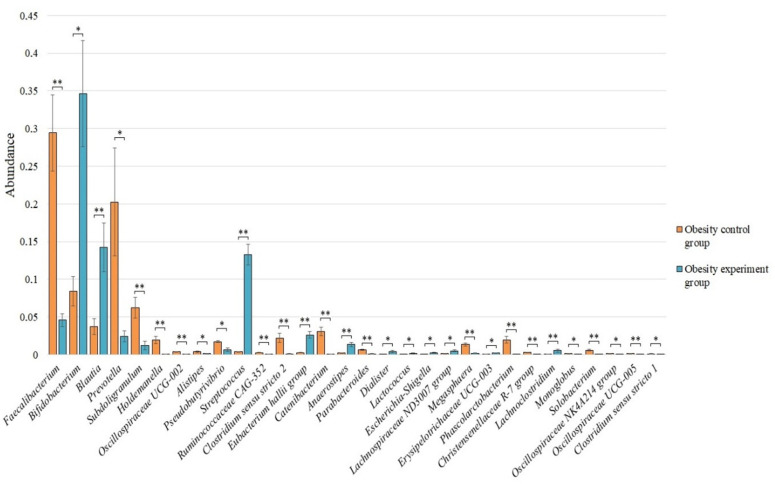
The statistically significant differences in the content of the genera of bacteria among the obesity control group and the obesity group of patients after the intake of lactic acid bacteria. * *p* < 0.05; ** *p* < 0.01.

Statistically significant differences in the microbiome composition before and after the intake of lactic acid bacteria by an obesity group of patients were identified for 30 bacterial genera (Figure 5).

After taking lactic acid bacteria, we observed a statistically significant increase in the genus *Bifidobacterium*—0.346 ± 0.070 vs. 0.084 ± 0.019 (*p* < 0.05), *Blautia*—0.142 ± 0.033 vs. 0.037 ± 0.011 (*p* < 0.01), *Streptococcus*—0.132 ± 0.014 vs. 0.004 ± 0.000 (*p* < 0.01), *Eubacterium hallii group*—0.026 ± 0.005 vs. 0.003 ± 0.001 (*p* < 0.01), *Anaerostipes*—0.013 ± 0.003 vs. 0.002 ± 0.001 (*p* < 0.01), *Dialister*—0.004 ± 0.001 vs. 0.000 ± 0.000 (*p* < 0.05), *Lactococcus*—0.002 ± 0.001 vs. 0.000 ± 0.000 (*p* < 0.05), *Escherichia-Shigella*—0.003 ± 0.001 vs. 0.000 ± 0.000 (*p* < 0.05), *Lachnospiraceae ND3007 group*—0.005 ± 0.001 vs. 0.001 ± 0.000 (*p* < 0.05), *Erysipelotrichaceae UCG-003*—0.002 ± 0.000 vs. 0.001 ± 0.000 (*p* < 0.05), and *Lachnoclostridium*—0.005 ± 0.002 vs. 0.001 ± 0.000 compared with the pre-supplement microbiome control obesity group, respectively.

At the same time, we observed a decrease in the number for the genus *Faecalibacterium*—0.046 ± 0.009 vs. 0.294 ± 0.051 (*p* < 0.01), *Prevotella*—0.024 ± 0.007 vs. 0.203 ± 0.072 (*p* < 0.05), *Subdoligranulum*—0.012 ± 0.006 vs. 0.062 ± 0.014 (*p* < 0.01), *Holdemanella*—0.000 ± 0.000 vs. 0.019 ± 0.005 (*p* < 0.01), *Oscillospiraceae UCG-002*—0.001 ± 0.000 vs. 0.004 ± 0.000 (*p* < 0.01), *Alistipes*—0.001 ± 0.000 vs. 0.004 ± 0.001 (*p* < 0.05), *Pseudobutyrivibrio*—0.006 ± 0.002 vs. 0.017 ± 0.001 (*p* < 0.05), *Ruminococcaceae CAG-352*—0.001 ± 0.000 vs. 0.003 ± 0.000 (*p* < 0.01), *Clostridium sensu stricto 2*—0.001 ± 0.000 vs. 0.022 ± 0.006 (*p* < 0.01), *Catenibacterium*—0.000 ± 0.000 vs. 0.031 ± 0.005 (*p* < 0.01), *Parabacteroides*—0.001 ± 0.000 vs. 0.007 ± 0.001 (*p* < 0.01), *Megasphaera*—0.002 ± 0.001 vs. 0.013 ± 0.002 (*p* < 0.01), *Phascolarctobacterium*—0.000 ± 0.000 vs. 0.020 ± 0.005 (*p* < 0.01), *Christensenellaceae R-7 group*—0.000 ± 0.000 vs. 0.003 ± 0.000 (*p* < 0.01), *Monoglobus*—0.000 ± 0.000 vs. 0.001 ± 0.000 (*p* < 0.05), *Solobacterium*—0.000 ± 0.000 vs. 0.006 ± 0.001 (*p* < 0.01), *Oscillospiraceae NK4A214 group*—0.000 ± 0.000 vs. 0.001 ± 0.000 (*p* < 0.05), *Oscillospiraceae UCG-005*—0.000 ± 0.000 vs. 0.001 ± 0.000 (*p* < 0.01), and *Clostridium sensu stricto 1*—0.000 ± 0.000 vs. 0.001 ± 0.000 (*p* < 0.05) after taking the lactic acid bacteria compared to the obesity control, respectively.

## 4. Discussion

### 4.1. Changes in Microbiome Composition at the Phylum Level

The phylum Actinobacteriota, whose increase we observed after the ingestion of lactic acid bacteria, includes members that can have a beneficial effect on intestinal health. It is also known that there is an inverse correlation of the number of this phylum with indicators characteristic of irritable bowel syndrome [25,26].

After 14 days of lactic acid bacteria intake, the number of the Bacteroidota phyla decreased. Members of this type are the main colonizers of the gastrointestinal tract. Many of these have the ability to produce butyrate, which is involved in maintaining gut health [27]. However, it is also known that an increase in individual members of Bacteroidota is associated with the development of obesity and inflammatory bowel disease [28,29].

### 4.2. Changes in the Relative Abundance of Beneficial Bacteria

In our study, we observed changes in the abundance of some potentially beneficial bacteria, particularly the genera *Bifidobacterium*, *Blautia*, *Eubacterium hallii group*, *Anaerostipes*, *Lactococcus,* and *Lachnospiraceae ND3007* group. In contrast, the number of representatives of some other beneficial genus (e.g., *Subdoligranulum*, *Holdemanella*, *Parabacteroides*) decreased after probiotics in obese patients.

Members of the genus *Bifidobacterium* are commensal microorganisms found in the human gastrointestinal tract and are traditionally considered beneficial to human health [30]. A study by Cerano C. Da Silva et al. showed that *Bifidobacterium* species were involved in the reduction of body weight and fat mass in Sprague-Dawley rats fed a high-fat diet [31]. This suggests that *Bifidobacterium* spp. potentially have anti-obesity properties.

Members of the genus *Blautia* are well-known as butyrate-producing bacteria. This metabolite may explain the beneficial role of these bacteria in glucose metabolism and inflammation associated with obesity [32,33].

*Eubacterium hallii group* is a butyrate-producing bacterium [34]. Unlike many other bacteria (e.g., *Roseburia*) that produce butyrate from monosaccharides, *E. hallii* can produce butyrate from lactate acetate in a low pH environment [35]. In a study by Shanthadevi Udayappan et al., the oral administration of active *E. hallii* improved the insulin sensitivity in severely resistant db/db mice insulin. In addition, *E. hallii* has been shown to contribute to changes in SCFA production and bile acid composition. The ability to exert these effects suggests that *E. hallii* is capable of beneficially affecting insulin sensitivity [32].

*Anaerostipes* is the most efficient consumer of lactate in the human colon [36]. It was previously shown that some bacteria of this genus are able to reduce glucose levels [37], and can also promote the production of propionate through inositol or phytate, thereby reducing the risk of metabolic diseases [38]. These data point to the potential health benefits of bacteria of the genus *Anaerostipes*.

The genus *Lactococcus* is a species of lactic acid bacteria that produce lactic acid as a result of the fermentation of carbohydrates [39]. Many species of *Lactococcus* are widely used as starter bacteria in the production of cheese and other fermented dairy products. However, there have been several studies showing the possibility of the presence of strains of *Lactococcus* in the flora of the gastrointestinal tract of humans or animals [40,41]. They are not pathogenic to humans, especially *Lactococcus lactis* and *Lactococcus garvieae*, which are able to produce antimicrobial agents [42]. Some species of bacteria of the genus *Lactococcus* including *Lactococcus lactis* and *Lactococcus petauri* have probiotic properties [43,44]. All of this suggests that the intake of probiotics based on lactic acid bacteria against the background of obesity contributes to an increase in the number of beneficial bacteria of the genus *Lactococcus*.

Members of the *Lachnospiraceae ND3007* group are putative producers of short-chain fatty acids (SCFAs) [45]. It has been shown that with a significant and inverse correlation, the *Lachnospiraceae ND3007* group has been associated with a decrease in the blood glucose levels in rats with diabetes [33].

It is known that bacteria of the genus *Subdoligranulum* are SCFA producers and are conditionally positive for the body [46]. It has also been previously noted that the genus *Subdoligranulum* is positively associated with overall metabolic health including lower body mass index and fat mass in adult humans [47].

The genus *Holdemanella* is considered to be a potential health contributor [48]. A positive correlation of the bacterial genus *Holdemanella* with the consumption of fermented dairy products, carbohydrates, and fiber was previously reported [49].

Members of the genus *Parabacteroides* are saccharolytic bacteria and produce major fermentation end products such as acetic acid and succinic acid. According to numerous studies, the relative abundance of *Parabacteroides* has been negatively associated with BMI [50,51,52,53] In addition, according to studies, some species such as *Parabacteroides goldsteinii* and *Parabacteroides distasonis* are considered as promising probiotics that may alleviate obesity and metabolic dysfunctions associated with obesity [53,54]. However, in our study, we observed a decrease in the abundance of this genus after the intake of lactic acid bacteria.

### 4.3. Changes in the Number of Pathogenic and Opportunistic Bacteria after Taking Probiotics

As a result of taking probiotics, we observed a decrease in some pathogenic bacteria such as *Clostridium sensu stricto 2* and *Solobacterium*. Furthermore, an increase in the relative abundance of the potentially pathogenic genus *Streptococcus* was observed.

Previously, the bacterial genus *Clostridium sensu stricto 2* was identified in human feces and clinical specimens such as blood, peritoneal and pleural fluid as well as lung biopsies from lung infections [55]. Members of this genus are associated with the development of infectious diseases: gas gangrene as well as mixed infections and metritis [56]. Thus, the data obtained indicate that this genus of bacteria has a high pathogenicity, which contributes to the development of infectious diseases.

*Solobacterium* is a Gram-positive obligate anaerobic genus in the family *Erysipelotrichidae* with one known species. This genus is part of the salivary microbiome, but is also found in small amounts in the colon [57,58], which refers to conditionally pathogenic microorganisms. Numerous studies have revealed a positive relationship between the increase in the abundance of this genus and the development of obesity [59,60]

Most *Streptococcus* species are considered pathogenic, but *Streptococcus thermophilus*, for example, is a probiotic [61]. *S. thermophilus* was in the composition of the final product containing lactic acid bacteria (see Materials and Methods). *S. thermophilus* is known to ferment lactose and sucrose, and can also metabolize fructose monosaccharide [62]. High fructose levels may be due to low numbers of these beneficial bacteria [63].

### 4.4. Changes in the Number of Bacteria Associated with Obesity

In our study, we found an increase in obesity-associated bacteria such as *Dialister* and *Lachnoclostridium*. Furthermore, some bacteria with a role associated with obesity decreased including *Faecalibacterium*, *Alistipes*, *Prevotella*, *Catenibacterium*, *Megasphaera*, *Phascolarctobacterium*, *Christensenellaceae R-7 group*, *Monoglobus*, and the *Oscillospiraceae NK4A214* group.

A study by Yanrong Lv et al. showed that the abundance of *Dialister* bacteria was lower in the overweight group compared to the lean group [64]. In our study, the intake of lactic acid bacteria led to an increase in the number of these bacteria in obese people. It is possible that bacteria of the genus *Dialister* play some role in the development of obesity, but more detailed studies of these bacteria are needed to understand the exact mechanisms.

In a study by Ana Nogal et al., increased levels of *Lachnoclostridium* bacteria led to lower levels of circulating acetate, which ultimately led to increased visceral fat. However, the study only looked at women and also only measured the changes in acetate, not all SCFAs [65]. We found that the relative abundance of members of the genus *Lachnoclostridium* increased in obese patients after taking probiotics. This suggests that more research is needed on the role of *Lachnoclostridium* bacteria in obesity.

Studies using the analysis of the association of individual microbial genera (according to *16S rRNA* gene sequencing) with obesity or type 2 diabetes mellitus showed that the reduced activity of *Faecalibacterium*, *Alistipes* is significant in relation to obesity [66]. This is consistent with the results obtained in our study for these genera of bacteria.

The genus *Alistipes* is known to be anaerobic bacteria found mainly in the microbiota of the gastrointestinal tract of healthy humans [67]. According to the results of studies, a marked decrease in the abundance of the genus *Alistipes* was observed in people with obesity compared with people with normal weight [66]. Additionally, the results were revealed in a study by Duan et al. (2021), where significant differences in the microbiota between people diagnosed with obesity and the control group, and the number of bacteria of the genus *Alistipes* significantly decreased [68]. In our study, a trend toward a decrease in the number of this genus of bacteria was obtained after two weeks of supplementation containing lactic acid bacteria. The differences between the obtained results and the literature data indicate an incomplete study of this genus of bacteria in relation to obesity.

*Prevotella* also decreased after two weeks of dietary lactic acid bacteria supplementation, and these results are consistent with the results of a study by Duan et al. (2021), where a significant difference was demonstrated between the obese and control group [68]. Hu H.J. et al. also reported an increase in the abundance of the genus *Prevotella* in people with obesity [69]. It has been established that bacteria of this genus are able to separate insoluble plant fiber and ferment soluble carbohydrates to form SCFAs [70]. The revealed ability of the genus *Prevotella* may be useful on a low-calorie diet, but in turn can lead to obesity on a fairly high-calorie diet. The results of studies demonstrate the association of the abundance of the *Prevotella* enterotype with a long-term diet that included a high intake of soluble carbohydrates and simple sugars and a low intake of proteins, amino acids, and saturated fats [71,72].

The study by Martínez-Cuesta et al. (2021) noted that bacteria of the genus *Catenibacterium* were significantly higher in obese participants, while the bacterial genus *Alistipes* was reduced [73].

*Megasphaera* is a genus of Gram-negative, non-motile, coccoid, opportunistic bacteria [74]. It is a part of the resident microflora of the human colon. This genus produces SCFAs: butyric, isobutyric, valeric, caproic, isovaleric, and isocaproic. This bacterium can use lactate and produce beneficial butyrate [75]. However, the pathway of its formation is associated with the production of ammonia, which can harm the host [76]. A direct correlation was found between the abundance of the genus and indicators characteristic of obesity [51,77,78].

*Phascolarctobacterium* is a relatively numerous genus of bacteria in the human gastrointestinal tract. A study in obese rats showed that an increase in *Phascolarctobacterium* was positively correlated with an increase in body weight, fat mass, plasma leptin, triglycerides, and glucose tolerance [79]. It is also associated with unfavorable glycemic metabolism in a healthy young population [80]. In addition, according to some data, this genus can be considered as a biomarker for type 2 diabetes mellitus [81].

Most members of the *Christensenellaceae R-7* group genus are saccharolytic, and they can ferment carbohydrates with the accumulation of a mixture of SCFAs [82]. Studies have also confirmed a negative correlation between the abundance of the *Christensenellaceae R-7* group and body weight and visceral fat content [82,83,84]. Although *Christensenellaceae R-7* is believed to be reduced in patients suffering from intestinal diseases, we noted a reduction in this genus with a probiotic mixture of lactic acid bacteria.

The genus *Monoglobus* is one of the main specialized taxa involved in the fermentation of pectin and mannan in the human colon [85,86]. There are few studies describing the role of the genus *Monoglobus* in the effect of metabolic disorders. It is known to be inversely correlated with BMI as well as with the development of ulcerative colitis [87,88].

According to the literature data, the abundance of the *Oscillospiraceae NK4A214* group genus has a direct correlation with obesity, regardless of the cohort studied, and therefore is a contender for one of the prognostic genera for obesity [89].

### 4.5. Changes in the Abundance of Bacteria Whose Role Is Ambiguous or Unknown

The role of some bacteria, the number of which changed in our study, is still poorly understood.

*Escherichia* and *Shigella* are two closely related genera that share bioenergetic mechanisms that allow them to fill a specific niche in the gut microbiome ecosystem [90]. The genus *Escherichia-Shigella* includes four *Escherichia* species, four *Shigella* species, and several lineages currently unclassified as “*Escherichia cryptic clades*” [91]. All of them have different functions, some species exhibit pathogenic properties, while others are described as beneficial to humans [92,93,94]. In our study, a limitation of the approach is that the type of assay used combines different species of bacteria of the genus *Escherichia-Shigella* with a wide range of functions. Thus, this does not allow us to draw clear conclusions about the role played by the increase in the relative abundance of bacteria of the genus *Escherichia-Shigella* in the case of obesity after the ingestion of lactic acid bacteria.

In our study, a reduction in bacteria belonging to the genus *Oscillospiraceae UCG-002* was demonstrated after two weeks of lactic acid supplementation. Bacteria of the genus *Oscillospiraceae UCG-002* are known to significantly contribute to aspartate degradation and glycine degradation [95].

The bacterial genus *Ruminococcaceae CAG-352* also showed a decrease after two weeks of lactic acid bacteria supplementation. The genus *Ruminococcaceae CAG-352* is known to be Gram-positive bacteria [96]. We did not find evidence of an association of this genus of bacteria with obesity in the literature.

For the genus *Oscillospiraceae UCG-005*, we observed a decrease in abundance after lactic acid bacteria supplementation, but the relationship of this genus with any metabolic pathologies in humans is poorly understood. Further research is needed to shed light on the contribution of this genus to gut health.

The contribution of *Clostridium sensu stricto 1* to human gut health is poorly understood. There are data that have found a direct correlation of *Clostridium sensu stricto 1* with acute pancreatitis in rats [97] and with necrotic enteritis [98]. It is also thought that an abundance of *Clostridium sensu stricto 1* may be responsible for the visceral hypersensitivity seen in irritable bowel syndrome [99]. Further studies are needed to suggest what role this bacterial cluster plays in the human gut. We found a reduction of this kind after the intake of lactic acid bacteria in obese patients.

Little is known about the role of bacteria of the genus *Erysipelotrichaceae UCG-003*, so it is difficult to say exactly what role members of this genus play.

## 5. Conclusions

The studied mixture of lactic acid bacteria, consisting of strains of *L. acidophilus VSUET12*, *L. plantarum VSUET13*, *L. fermentum VSUET14*, and *Lactobacillus delbrueckii subsp bulgaricus VSUET15*, which was taken by obese people, had a significant effect on changing the gut microbiome landscape. According to the results of the study, we observed at the phylum level, a decrease in Bacteroidota and an increase in Actinobacteriota, both of which belong to the main phylum that form the core of the microbiome. At the generic level, a 14-day intake of the mixture of lactic acid bacteria led to the enrichment of the potentially beneficial groups *Bifidobacterium*, *Blautia*, *E. halli* group, *Anaerostipes*, *Lactococcus*, and *Lachnospiraceae ND3007*. We also observed an increase in the genera *Streptococcus*, *Escherichia-Shigella*, and *Lachnoclostridium*, which contributed to gut health and is highly member-dependent.

We also found a decrease in the genera *Faecalibacterium, Pseudobutyrivibrio, Subdoligranulum, Faecalibacterium, Clostridium sensu stricto 1* and *2*, *Catenibacterium, Megasphaera, Phascolarctobacterium, Oscillospiraceae NK4A214 group*, which can be associated with the development of various metabolic disorders as well as inflammatory bowel diseases. At the same time, the abundance of beneficial genera such as *Subdoligranulum, Holdemanella, Alistipes, Parabacteroides, Christensenellaceae R-7, Monoglobus*, and *Solobacterium* also decreased. The decrease in the genera *Prevotella, Oscillospiraceae UCG-002*, and *UCG-005, Ruminococcaceae CAG-352* cannot be interpreted unambiguously. The effect of some of them on the host organism depends on the specific species and strain, while others are poorly understood.

Thus, based on the data obtained, we cannot unambiguously conclude on the beneficial effect of the mixture of the studied lactic acid bacteria on the gut of obese people. Positive microbiome changes were observed against the background of the intake, but along with these, there were changes in the gut microbiota that could not be interpreted as positive.

## Figures and Tables

**Table 1:** The primers used in the study.

Name of the Primer	Primer Sequence
337F	5′-GACTCCTACGGGAGGCWGCAG-3′
518R	5′-GTATTACCGCGGCTGCTGG-3′

**Table 2:** The PCR protocol for amplification of the V3 hypervariable region of the *16S rRNA* gene.

Stage Name	Temperature	Time	The Number of Cycles
General denaturation	94 °C	4 min	1
Denaturation	94 °C	30 s	37
Primer annealing	53 °C	30 s	
Elongation	72 °C	30 s	
Final elongation	72 °C	5 min	1

## Data Availability

Sequencing data are available in the NCBI BioProject database (BioProject: PRJNA862929).

## Supplementary Materials

The following supporting information can be downloaded at: https://www.mdpi.com/article/10.3390/nu14163384/s1, Table S1: The abundance of microorganisms for the samples.
